# Factors associated with access to HIV care and treatment in a prevention of mother to child transmission programme in urban Zimbabwe

**DOI:** 10.1186/1758-2652-13-38

**Published:** 2010-10-06

**Authors:** Auxilia Muchedzi, Winfreda Chandisarewa, Jo Keatinge, Lynda Stranix-Chibanda, Godfrey Woelk, Elizabeth Mbizvo, Avinash K Shetty

**Affiliations:** 1 Zimbabwe AIDS Prevention Project-University of Zimbabwe, Department of Community Medicine, Zimbabwe; 2 Elizabeth Glaser Pediatric AIDS Foundation Country Office, Harare, Zimbabwe; 3 UZ-UCSF Collaborative Program in Women's Health, Harare, Zimbabwe; 4 Ministry of Health and Child Welfare, Harare, Zimbabwe; 5 Department of Paediatrics, Wake Forest University Health Sciences, Winston-Salem, NC, USA

## Abstract

**Background:**

This cross-sectional study assessed factors affecting access to antiretroviral therapy (ART) among HIV-positive women from the prevention of mother to child transmission HIV programme in Chitungwiza, Zimbabwe.

**Methods:**

Data were collected between June and August 2008. HIV-positive women attending antenatal clinics who had been referred to the national ART programme from January 2006 until December 2007 were surveyed. The questionnaire collected socio-demographic data, treatment-seeking behaviours, and positive or negative factors that affect access to HIV care and treatment.

**Results:**

Of the 147 HIV-positive women interviewed, 95 (65%) had registered with the ART programme. However, documentation of the referral was noted in only 23 (16%) of cases. Of the 95 registered women, 35 (37%) were receiving ART; 17 (18%) had not undergone CD4 testing. Multivariate analysis revealed that participants who understood the referral process were three times more likely to access HIV care and treatment (OR = 3.21, 95% CI 1.89-11.65) and participants enrolled in an HIV support group were twice as likely to access care and treatment (OR = 2.34, 95% CI 1.13-4.88). Those living with a male partner were 60% less likely to access care and treatment (OR = 0.40, 95% CI 0.16-0.99). Participants who accessed HIV care and treatment faced several challenges, including long waiting times (46%), unreliable access to laboratory testing (35%) and high transport costs (12%). Of the 147 clients surveyed, 52 (35%) women did not access HIV care and treatment. Barriers included perceived long queues (50%), competing life priorities, such as seeking food or shelter (33%) and inadequate referral information (15%).

**Conclusions:**

Despite many challenges, the majority of participants accessed HIV care. Development of referral tools and decentralization of CD4 testing to clinics will improve access to ART. Psychosocial support can be a successful entry point to encourage client referral to care and treatment programmes.

## Background

Zimbabwe has one of the highest HIV infection rates in the world [1-3]. In 2007, the HIV prevalence among pregnant women attending antenatal clinics was around 15.6% [2,3]. Prevention of mother to child transmission (PMTCT) of HIV is a major public health challenge in Zimbabwe [4]. Linking PMTCT programmes with HIV care and treatment is critical for promoting maternal health and survival and to reduce paediatric HIV infection [4]. Although scale up of antiretroviral therapy (ART) is occurring in most countries in sub-Saharan Africa, data indicate that less than 10% of HIV-infected pregnant women in sub-Saharan Africa have access to ART [5].

The Zimbabwe AIDS Prevention Project (ZAPP)-Family AIDS Initiatives (FAI) supports the implementation of the national PMTCT programme at five antenatal sites in Chitungwiza. Since 2002, the ZAPP-FAI programme has been funded by the Elizabeth Glaser Pediatric AIDS Foundation (EGPAF) and United States Agency for International Development (USAID) [3]. In 2004, the Government of Zimbabwe started to roll out the ART programme at several sites across the country, including Chitungwiza [2].

In low-income countries, traditionally PMTCT and HIV care and treatment programmes have been running as parallel programmes with weak linkages [6]. Recent studies from resource-limited settings have shown that the majority of HIV-infected women and their partners face considerable challenges in accessing care, treatment and support services [7-12]. There is very limited research on barriers to access HIV care and treatment among HIV-positive pregnant women from the PMTCT programme in sub-Saharan Africa [13,14]. To our knowledge, there is no published literature on this topic from Zimbabwe. The objective of this study was to assess the factors affecting access to ART and HIV care among HIV-positive women who participated in a PMTCT programme in urban Zimbabwe.

## Methods

### Study design, setting and sample

Between June and August 2008, a cross-sectional study of factors affecting access to ART and HIV care among HIV-positive women from the PMTCT programme was performed. The study was conducted at the four municipal antenatal clinics in Chitungwiza, a high-density urban town in Zimbabwe with a population of 1.2 million. Approximately 10,000 pregnant women book for antenatal care annually in the Chitungwiza clinics.

A basic package of PMTCT services was provided at the antenatal clinics, including training of healthcare workers on PMTCT, routine antenatal HIV counselling and testing ("opt-out" approach) for all pregnant women using rapid HIV testing, administration of single-dose mother/infant nevirapine (NVP) regimen, counselling and support on infant feeding choices according to World Health Organization (WHO) guidelines, establishment of community-based psychosocial support groups, and mother-infant follow up until 18 months after delivery [3]. Overall, the uptake of single-dose mother/infant NVP uptake exceeds 90% in the clinics.

The study population included all HIV-positive women from the PMTCT programme at the antenatal clinics who were referred to the national ART programme at the Chitungwiza Central Hospital from January 2006 until December 2007. The referral was made based on symptomatic disease and/or clinic physician's assessment of mothers based on WHO clinical staging during antenatal/post-partum follow-up visits. At the antenatal clinics, CD4 cell count testing facilities were unavailable at the time of data collection. During the referral counselling sessions, peer counsellors (also referred to as "community mobilizers") inform the clients regarding the registration process at the ART clinic at the hospital.

Documentation of the referral between the PMTCT programme at the antenatal clinic and the ART programme at the hospital is done by a two-step referral form. After counselling, peer counsellors at the clinic (referring end) complete the top part of the referral form and document the referral made in a "registration book" kept at the clinic. The clients then take the referral form to the ART clinic at the hospital (receiving end). During registration, the healthcare worker in the ART clinic completes and retains the bottom part of the referral form in a folder, while the top part of the referral form is returned to the client to take back to the PMTCT clinic for review by the clinic physician/peer counsellors, who then document the completed referral in the registration book.

### Recruitment procedures

The clinic offers a unique mentorship programme led by peer counsellors ("community mobilizers"). HIV-infected women who had had previously participated in a PMTCT programme at our site, were currently enrolled in support groups, and had disclosed their positive HIV status to partners or family members were selected to become peer counsellors [3]. The peer counsellors are assigned to the antenatal and postnatal clinics and trained to deliver health education talks to antenatal women, provide psychosocial support and counsel mothers on disclosure and infant feeding, facilitate mother-infant follow up, and engage in community mobilization. The peer counsellors also conduct home visits in the community if the clients fail to show up for their appointments.

Study participants were identified and recruited for interviews by peer counsellors at postnatal clinics, PMTCT support group meetings and the infant follow-up clinic. HIV-positive women who were participating in other ongoing research studies at the clinics that facilitated transportation and registration at the ART clinic were excluded from this study. Clients who came for ANC bookings while they were already on ART were also excluded, as were male partners and HIV-infected infants and children referred for care and treatment services.

### Data collection

After informed consent, a pre-tested structured questionnaire was administered to all eligible participants in the local language, Shona, by four trained bilingual research assistants. The interviews were conducted in a private room in the clinic and lasted for approximately 45 minutes. Clients who did not follow up to attend clinic-based PMTCT activities within the first four weeks of study were visited at their homes by peer counsellors and invited to come to the clinic to participate in the study. No incentives were provided for the mothers to participate in the interviews.

### Development of study measure

Two focus group discussions (FGDs) were conducted with groups of 10 to 12 clients from the PMTCT programme at the Highfield polyclinic in Harare and key informants in the antenatal clinic to explore the factors associated with accessing HIV care and treatment and develop the study questionnaire. The Highfield clinic is located in the capital city of Harare, which has a similar demographic and socioeconomic status to Chitungwiza. The FGDs identified themes to be included during the interviews with clients. They also ensured that the language and phraseology used on the questionnaire and consent forms were appropriate and interpreted correctly by the community. A pre-set discussion guide was used during the FGDs, with one of the authors (AM) serving as a facilitator. The discussions were conducted in the local language of the group, taped, translated and transcribed by the facilitator for analysis. The narratives from the FGDs were analyzed using a two-stage thematic analysis. The data generated from the FGDs were used to develop the study questionnaire.

### The study questionnaire

From the FGDs, a structured questionnaire, which had both open-ended and closed-ended questions, was developed. The questionnaire collected socio-demographic data, treatment-seeking behaviours, and positive or negative factors that affect access to HIV care and treatment. In this study "access to HIV care and treatment" was defined as successful completion of registration at the ART clinic. The questionnaire was reviewed for both the content and structure by all the authors. It was then translated into Shona and then back into English by a local bilingual translator to be sure the intended meaning was conveyed.

### Ethical considerations

This study was approved by the Medical Research Council of Zimbabwe and the Institutional Review Board at Wake Forest University Health Sciences. Ethical approval was also obtained from the Chitungwiza Health Department and the Zimbabwe Ministry of Health and Child Welfare. Informed consent was obtained from all participants.

### Statistical analysis

Data was entered using the Epi Info 2002 version 3.2.3 (Centers for Disease Control and Prevention, Atlanta, GA) and analyzed using STATA version 10 (STATA Corp, College Station, TX, USA). Continuous variables were summarized using medians (interquartile range) and categorical variables using proportions. Groups were compared for differences using the Chi-square test for categorical variables and Mann-Whitney U test for continuous variables. Logistic regression was used to identify the barriers and enhancers of access to HIV care among HIV-infected women. All variables that yielded a p-value of <0.2 in the univariate analysis were tested in the multivariate model. The results of the logistic regression analyses are depicted as odds ratio (ORs) and their corresponding 95% confidence intervals (CIs). We considered an association to be statistically significant if p ≤0.05.

## Results

**Figure 1:**
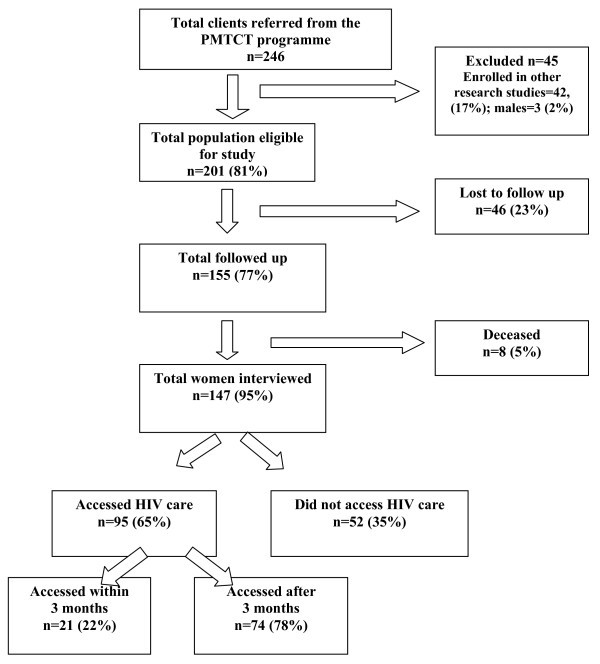
**Study profile**.

A total of 246 HIV-infected women from the PMTCT programme were referred to the ART programme at the Chitungwiza Central Hospital between January 2006 and December 2007. Figure 1 shows the study profile. The majority of the participants (66%) were recruited by peer counsellors at the postnatal clinic, followed by support group meetings (25%), the infant follow-up clinic (7%) and at home (3%). None of the eligible participants refused to participate.

Of the 246 participants referred, 45 (19%) women were excluded from the study because they were participating in a US National Institutes of Health-funded clinical trial, which facilitated transportation and registration of clients at the ART programme. Of the 201 eligible participants, 46 (23%) were lost to follow up, while eight (4%) had deceased by the time of the interviews. Of the 46 clients who were lost to follow up, 41 (20%) had moved out of the study area, while five (10%) had relocated to other countries in the region.

Of the 147 participants who were interviewed and eligible for inclusion in this survey, 95 (65%) had registered with the ART programme, while 52 (35%) had not registered. However, on review of the registration book at the antenatal clinic, documentation of the referral between the PMTCT programme and the ART clinic was noted in 23 (16%) of cases.

### Socio-demographic characteristics

Table 1 summarizes the socio-demographic characteristics of the study participants. There were no significant differences in the demographic characteristics of the participants who accessed HIV care and those who did not access care. The mean age of the participants who accessed care and treatment was 30.1 ± 5.1 years compared with the mean age of 29.1 ± 5.1 years among those who did not access care and treatment. The majority of the participants were married (78%), had attained secondary education (83%) and were self-employed (58%). The main source of household income was the male partner (57%). More than a third of the study participants (35%) belonged to the Apostolic Faith (a unique religious sect in Zimbabwe, which does not believe in modern medicine). The majority (66%) of the participants lived within walking distance to Chitungwiza Central Hospital, where the ART clinic is located, whereas 27% of the participants used public transport to get to the ART clinic.

**Table 1:** Demographic characteristics of participants (N = 147)

Characteristics	Total number	Accessed HIV care	Accessed HIV care (%)
**Marital status**			
Married	114	70	61
Widowed	18	14	78
Divorced	11	8	73
Single	4	3	75
**Educational level**			
Secondary	122	82	67
Primary	23	11	48
Tertiary	2	2	100
**Employment status**			
Self-employed	85	55	65
Unemployed	54	33	61
Formally employed	8	7	87
**Religion**			
Apostolic	51	34	67
Pentecostal	32	21	66
Catholic	22	17	77
Methodist	13	7	54
Anglican	10	5	50
Seventh Day Adventist	7	5	71
Other	12	6	50
**Source of income**			
Husband	83	53	64
Self	48	33	69
Parents	4	2	50
Others	12	7	58
**Transport to ART clinic**			
Walking	97	66	68
Public transport	40	22	55
Walk or public transport	9	7	78
Own transport	1	0	0

ART, antiretroviral therapy

### Access to HIV care

Of the 147 participants interviewed, 95 (65%) had registered with the ART clinic. Of the 95 women who had registered, 35 (37%) participants had been initiated on ART; 17 (18%) had not undergone CD4 count testing; eight (8%) had CD4 counts of <350 cells/mm^3 ^but were not receiving ART, while 35 (37%) had CD4 counts of ≥350 cells/mm^3 ^and were monitored every six months for eligibility of ART. Of the 95 participants who were successfully registered with the ART programme, 21 (22%) made the first visit to the ART clinic after three months of being referred (median of 21 days Q1 = 7 days, Q3 = 120).

### Challenges faced by participants who accessed HIV care and treatment

Table 2 shows the challenges perceived by study participants who accessed HIV care and treatment services (n = 95). Participants who accessed HIV care and treatment faced several challenges, including: long waiting times at the ART clinic (46%); unreliable access to laboratory tests, such as CD4 count, complete blood counts and liver function tests (35%); and high transport costs (12%). The median duration of time spent by the participants at each visit to the ART clinic was reported to have been five hours (Q1 = 1 hour, Q4 = 10 hours). The interviewer asked the participants to report the duration of every single clinic visit that they had made, and then the median was calculated from the total of all visits made by all women.

**Table 2:** Challenges faced by HIV-positive women from the PMTCT programme who accessed HIV care and treatment services (n = 95)

Challenges	Frequency (%)
Long queues at the ART clinic	44 (46.3)
Constant breakdown of laboratory equipment	33 (34.7)
ART clinic too congested with very sick people	16 (16.8)
Transport for repeat visits too high	11 (11.6)
Service too slow, maybe due to staff shortages	8 (8.4)
Shortage of antiretroviral drugs	5 (5.2)
Registration process too long	5 (5.2)
Hospital administration fees unaffordable	4 (3.8)
Loss of confidentiality	3 (3.2)
Stigmatization by health workers	3 (3.2)
Fear that child may get opportunist infections at the ART clinic	2 (2.1)

*This was a multiple response question and hence the percentages adds up to more than 100%

ART, antiretroviral therapy; PMTCT, prevention of mother to child transmission of HIV

### Challenges faced by participants who did not access HIV care and treatment

Of the 147 clients surveyed, 52 (35%) women did not access HIV care and treatment. As shown in Table 3, reasons cited by women who did not access HIV care and treatment included the perception of a tedious registration process and/or long queues (50%), competing life priorities (33%) (e.g., seeking food or shelter, caring for other children in the home and caring for sick relatives), and inadequate referral information (15%).

**Table 3:** Reasons cited by HIV-positive women for not accessing HIV care and treatment services (n = 52)

Reasons	Frequency (%)
Perceived long queues/laborious registration process	26 (50)
Competing priorities	17 (32.7)
Inadequate referral information	8 (15.4)
High transport costs	7 (13.5)
Procrastination	4 (7.7)
Lost referral letter	3 (5.8)
Lack of disclosure	2 (3.8)
Did not believe the results	2 (3.8)
Fear loss of confidentiality	2 (3.8)
Fear side effects	1 (1.9)
High hospital fees	1 (1.9)

*This was a multiple response question and hence the percentages adds up to more than 100%

### Factors associated with access to HIV care and treatment

In the univariate analysis (Table 4), understanding the referral process was significantly associated with access to HIV care and treatment (OR = 3.09, CI 2.07-14.89), with those participants who understood the referral process being three times more likely to access HIV care and treatment than those who did not understand the referral process. Participants who were members of a HIV support group were twice as likely to access HIV care and treatment as those who did not belong to any HIV support group (OR = 2.39, CI 1.13-5.12).

**Table 4:** Univariate analyses for factors associated with access to HIV care and treatment among HIV-positive women from the PMTCT programme

Predictors	Unadjusted odds ratio	95% CI
**Understood the referral process**		
No	1	
Yes	3.09	2.07-14.89
**Joined any support group**		
No	1	
Yes	2.39	1.13-5.12
**Ever been stigmatized**		
No	1	
Yes	1.90	0.59-6.20
**Believed/accepted HIV status**		
No	1	
Yes	1.85	0.89-3.82
**Ready to be referred to ART clinic**		
No	1	
Yes	1.81	0.71-4.62
**Number of children who died**		
None	1	
At least one	1.40	0.48-2.05
**Witnessed someone being stigmatized**		
No	1	
Yes	1.38	0.65-2.93
**Feeling after being referred**		
Negative	1	
Positive/good	1.25	0.20-7.74
**Educational level**		
Secondary/tertiary	1	
Primary	0.44	0.17-1.12
**Living with male partner**		
No	1	
Yes	0.46	0.19-1.99
**Age group**		
> = 35	1	
<35	0.57	0.19-1.66
**PMTCT knowledge levels**		
> = 51%	1	
<51%	0.58	0.29-1.15
**Herbal treatments for HIV infection**		
No	1	
Yes	0.67	0.34-1.34
**Transport to ART clinic**		
Walk	1	
Public transport	0.69	0.32-1.49
**Household income**		
Self	1	
Partner	0.76	0.36-1.61
**Employment status**		
Employed	1	
Unemployed	0.795	0.40-1.59

ART, antiretroviral therapy; PMTCT, prevention of mother to child transmission of HIV

Multivariate analysis (Table 5) revealed that access to HIV care and treatment were significantly associated with participants who understood the referral process (OR = 3.21, CI 1.89-11.65) and those enrolled in an HIV support group (OR = 2.34, CI 1.13-4.88). Those living with a male partner were 60% less likely to access care and treatment (OR = 0.40, CI 0.16-0.99).

**Table 5:** Multivariate analyses for factors associated with access to HIV care and treatment among HIV-positive women from the PMTCT programme

Predictors	Adjusted odds ratio	95% CI
**Understood referral process**		
No	1	
Yes	3.21	1.89-11.65
**Joined any support group**		
No	1	
Yes	2.34	1.13-4.88
**Living with male partner**		
No	1	
Yes	0.40	0.16-0.99
**Educational level**		
Secondary/tertiary	1	
Primary	0.47	0.17-1.28
**Knowledge level of PMTCT**		
≥51%	1	
<51%	0.53	0.25-1.12
**Believed/accepted HIV status**		
No	1	
Yes	1.97	0.90-4.32

PMTCT, prevention of mother to child transmission of HIV

## Discussion

Very few studies from sub-Saharan Africa countries have described barriers to access care and treatment among HIV-positive pregnant women from the PMTCT programme [13,14]. This study is among the first to assess the factors affecting access to ART and HIV care among PMTCT women in Zimbabwe. We found that two-thirds of all PMTCT clients referred to the ART clinic at Chitungwiza Central Hospital between January 2006 and December 2007 accessed HIV care. However, documentation of the referral system between the PMTCT programme at the antenatal clinic and the ART programme at the hospital was poor (noted in only 16% of cases). The severe shortage of healthcare workers due to staff attrition at the ART clinic may have contributed to this finding. Development of referral tools (e.g., referral forms, registers) and strengthening the follow-up system could facilitate the link between PMTCT and ART programmes. The implementation of simple referral system using referral forms has been effective in linking diagnosed patients with ART clinics in rural Tanzania [15].

In the present study, even though 65% of HIV-infected women from the PMTCT programme had registered at the ART clinic, only 37% were receiving ART. The majority of PMTCT clients were at various stages in the process of accessing HIV care and treatment; 18% of registered women had not had their CD4 tested and 8% of participants with CD4 counts of <350 cells/mm^3 ^were not receiving ART. This finding is consistent with other reports from sub-Saharan Africa [5,7,8,10,12].

In our study, 22% of participants reported a delay of more than three months after a referral was made in accessing HIV care despite the fact that most of the clients were within walking distance of the ART clinic. This finding is of great concern since timely access to ART is critical for HIV-infected pregnant women with symptomatic disease to reduce the risk of mother to child transmission of HIV and promote maternal health and survival [16]. In addition, access to ART by post-partum mothers can reduce the risk of HIV transmission during the breastfeeding period [17].

Many challenges were reported by study participants who accessed HIV care and treatment services at the ART clinic. Nearly half of participants experienced long waiting times at the clinic. Waiting times is an important factor for patient dissatisfaction while accessing HIV care and treatment services [10,18]. In one study from Uganda, innovative organizational changes, including nurse visits and pharmacy-only refill visits, have been successful in significantly decreasing the waiting times at the clinic [19]. Other challenges cited include unreliable access to laboratory diagnostics, e.g., CD4 testing, and overcrowding at the ART clinics. Similar findings have been reported from Mozambique and Zambia [10,12].

In the Chitungwiza urban setting, most (66%) of the participants could walk to the ART clinic from their homes. In contrast, the long distance from the home to the ART clinic has been cited as a major barrier in many other settings in sub-Saharan Africa [10,17,20-22]. In rural settings, transport costs could be a major factor affecting access to HIV care [21,23]. Decentralization of HIV care and treatment services, including CD4 count testing, to clinic level will improve access to ART in our setting.

In this study, several barriers were reported by participants who did not access HIV care and treatment at the clinic. One-third of participants reported competing life priorities, such as earning livelihoods and seeking food and shelter, as major barriers to accessing HIV care. This finding is not surprising given the current economic crisis in Zimbabwe [24]. In contrast to other studies [25-27], stigma associated with HIV/AIDS did not discourage the study participants from accessing HIV care. More than two-thirds of the study participants were members of a PMTCT psychosocial support group, which may have promoted positive health-seeking treatment behaviours and effective strategies to cope with HIV-related stigma [28]. In this study, participants who were members of an HIV support group were twice as likely to access HIV care and treatment services as those that were not members of a support group.

This study also identified health system barriers facing participants who did access HIV care and treatment services. Perceived long queues at the clinic were reported as a major barrier by 50% of these participants. Inadequate referral information was reported by 15% of participants who did not access HIV care. Similar health system barriers have been reported in other studies conducted in developing countries [25,29]. Participants who reported that they had understood the referral process were three times more likely to access HIV care than those who did not access care.

It is recommended that information, education and communication materials should be developed to strengthen understanding of the referral systems. During the referral counselling sessions, peer counsellors can educate the clients regarding the registration process at the ART clinic using these materials. Given the low proportion of referrals that were documented in this study, it is also likely that healthcare workers were not able to accurately explain the referral process to the clients. Therefore, training and ongoing supervision of health workers is recommended. This is particularly important in our setting given the high staff attrition rates due to economic instability [24].

This study has several limitations. First, 23% of the participants were lost to follow up due to the mobility of the population in the city of Chitungwiza. Second, participants were not asked to rank barriers based on their relative importance. This makes it difficult to conclude which of the barriers identified was the most challenging to overcome. Third, this study was conducted in an urban setting and our study results may not potentially be generalized to rural settings. Finally, our study relied on self-report with a potential for reporting bias. Despite these limitations, our study provides significant insights into factors that affect access to HIV care and treatment and informs policy makers to find ways to address these barriers and encourage individuals to access treatment.

## Conclusions

Despite many challenges, the majority of women accessed HIV care following a referral from the PMTCT programme. Development of referral tools and decentralization of CD4 count testing and ART initiation within maternal and child health units may improve access to ART. In addition, psychosocial support can be a successful entry point to encourage referrals into care and treatment programmes.

## Competing interests

The authors declare that they have no competing interests.

## Authors' contributions

AM participated in the design, supervised study implementation and drafted the manuscript. AM, WC and LS participated in study implementation and data collection. AM, JK, GW and AS participated in data analysis and provided technical expertise. AM, WC, JK, EM and AS conceived the study, and participated in its design and coordination. All authors read and approved the final manuscript.
